# Antineoplastic effects of the DNA methylation inhibitor hydralazine and the histone deacetylase inhibitor valproic acid in cancer cell lines

**DOI:** 10.1186/1475-2867-6-2

**Published:** 2006-01-31

**Authors:** Alma Chavez-Blanco, Carlos Perez-Plasencia, Enrique Perez-Cardenas, Claudia Carrasco-Legleu, Edgar Rangel-Lopez, Blanca Segura-Pacheco, Lucia Taja-Chayeb, Catalina Trejo-Becerril, Aurora Gonzalez-Fierro, Myrna Candelaria, Gustavo Cabrera, Alfonso Duenas-Gonzalez

**Affiliations:** 1 Unidad de Investigación Biomédica en Cancer. Instituto de Investigaciones Biomédicas, UNAM, /Instituto Nacional de Cancerología, Mexico City, Mexico; 2 Division of Clinical Research, Instituto Nacional de Cancerología, Mexico City, Mexico

## Abstract

**Background:**

Among the epigenetic alterations occurring in cancer, DNA hypermethylation and histone hypoacetylation are the focus of intense research because their pharmacological inhibition has shown to produce antineoplastic activity in a variety of experimental models. The objective of this study was to evaluate the combined antineoplastic effect of the DNA methylation inhibitor hydralazine and the histone deacetylase inhibitor valproic acid in a panel of cancer cell lines.

**Results:**

Hydralazine showed no growth inhibitory effect on cervical, colon, breast, sarcoma, glioma, and head & neck cancer cell lines when used alone. On the contrary, valproic acid showed a strong growth inhibitory effect that is potentiated by hydralazine in some cell lines. Individually, hydralazine and valproic acid displayed distinctive effects upon global gene over-expression but the number of genes over-expressed increased when cells were treated with the combination. Treatment of HeLa cells with hydralazine and valproic acid lead to an increase in the cytotoxicity of gemcitabine, cisplatin and adriamycin. A higher antitumor effect of adriamycin was observed in mice xenografted with human fibrosarcoma cells when the animals were co-treated with hydralazine and valproic acid.

**Conclusion:**

Hydralazine and valproic acid, two widely used drugs for cardiovascular and neurological conditions respectively have promising antineoplastic effects when used concurrently and may increase the antitumor efficacy of current cytotoxic agents.

## Background

Modulation in the expression of cancer-related genes silenced by changes in DNA methylation and histone post-translational modification through the use of DNA methylation and histone deacetylase inhibitors has been shown to exert antitumor effects in *in vitro *and *in vivo *models [1]. These results have led to the development of these class of drugs for clinical use. The classical demethylating agents comprise analogs of deoxycytidine: 5-azacytidine, 5-aza-2-deoxycytidine, 1-β-D-arabinofuranosil-5-azacytosine, and dihydro-5-azacytidine, developed over the past 30 years as classical cytotoxic agents, and subsequently discovered as DNA methylation inhibitors [2]. Another class of demethylating agent is the antisense oligonucleotide MG98 directed against the3' untranslated region of *DNMT1 *mRNA, which codes for the enzyme DNA methyltransferase 1 responsible for maintenance of DNA methylation [3]. The fact that deoxycytidine analogs such as 5-aza-2-deoxycytidine are not only carcinogenic but also exhibit neutropenia as their dose-limiting toxicity when used for demethylation [4] has renewed interest in finding effective and less toxic demethylating agents.

Among these are an oral cytidine analog zebularine [5] and the green tea major polyphenol (-)-epigallocatechin-3-gallate [6]. Our group has recently shown *in vitro *and *in vivo *promoter demethylation and tumor suppressor gene transcriptional reactivation mediated by the antihypertensive compound hydralazine, a well-tolerated drug devoid of the common side effects of cytotoxic chemotherapy agents [7] and whose DNA demethylating activity can be explained by the interaction between its nitrogen atoms with residues Lys162 and Arg240 of the DNA methyltransferase active site, as shown in a silico model [8]. Its demethylating and gene reactivating activity were also shown in a phase I trial [9].

On the other hand, HDAC inhibition has been reported to induce tumor cell differentiation, apoptosis, or growth arrest, depending on the experimental system [10,11], and to sensitize cells to chemotherapy [12] or radiation therapy [13]. Currently, there are six structurally distinct classes of HDAC inhibitors at diverse stages of preclinical and clinical development. Valproic acid, an 8-carbon, branched-chained fatty acid well-known as an effective antiepileptic drug [14] belongs to the *Small molecular weight carboxilates *[15]. This drug causes hyperacetylation of the N-terminal tails of histones H3 and H4 *in vitro *and *in vivo *and inhibits HDAC activity, most probably by binding to the catalytic center and thereby blocking substrate access [16,17]. In contrast to other HDAC inhibitors, valproic acid has a good tolerability and safety profile as demonstrated by 35 years of use as a chronic therapy for epileptic disorders [14]. Its ability to inhibit deacetylase activity in solid tumors has recently been demonstrated [18]. We thus investigated if the use of both compounds in combination displayed an increased antineoplastic effect.

## Results

### Inhibitory activity of hydralazine and valproic acid in vitro

**Figure 1:**
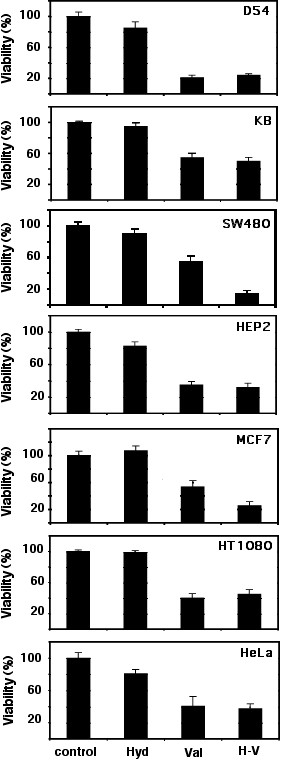
**In vitro growth inhibition by hydralazine, valproic acid and the combined treatment**. Hydralazine had not growth inhibitory effects at the dose and conditions tested in none of the cell lines. Valproic acid induced a variable degree of inhibition in the cell lines. D54 glioma cell line had 80% growth inhibition whereas KB laryngeal carcinoma was the least but still inhibition was close to 50%. MCF-7 and SW480 were more sensitive to the combination of compounds although the difference was statistically significant only in SW480 cells (p < 0.001)

Cells lines from different tumor types were treated with either hydralazine, valproic acid or the combination at 10 μM and 1 mM respectively for five days. As shown in Figure 1 all cell types were statistically significant growth inhibited to various degrees by valproic acid. D54 glioma cell line had 80% growth inhibition whereas KB laryngeal carcinoma was the least but still inhibition was close to 50%. On the other hand, hydralazine had no growth inhibitory effect at the dose and conditions tested. When used in combination, five of the seven cell lines showed the same inhibition that valproic acid alone. Interestingly, MCF-7 and SW480 were more sensitive to the combination of compounds although the difference was statistically significant only in SW480 cells (p < 0.001).

### Gene expression

**Figure 2:**
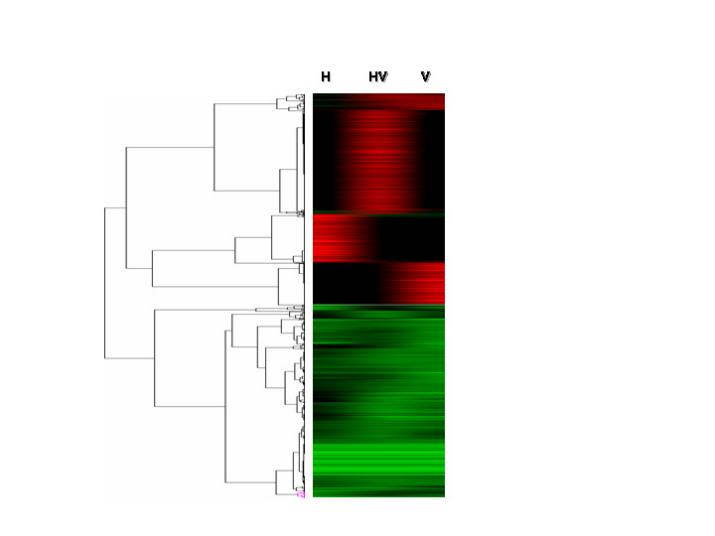
**Hierarchical clustering revealed specific patterns induced by each treatment**. Hydralazine, valproic acid and the combination down-regulated a similar set of genes. On the contrary, up-regulated genes were distinctive for each treatment. From 1,281 differentially expressed genes, 651 were up-regulated and 630 down-regulated. The number of genes over-expressed specific of hydralazine, valproic acid and the combination were 153, 178 and 352. Each row represents a gene, whereas each column corresponds to the treatment drug. The relative abundance of the gene in the tissue correlates with color intensity; red induced; green repressed; black no change. H: hydralazine, V: valproic acid, and HV: hydralazine and valproic acid.

To analyze any change in the global pattern of gene expression induced by these epigenetic active drugs, genome wide expression analysis was performed following drug treatment as described in methods section. RNA purified from SW480 was hybridized on the CodeLink system (Amersham; Piscataway, NJ). This array contains a broad range of genes derived from publicly available, well-annotated mRNA sequences (over 55,000 genes and ESTs probes). Following normalization and log2 transformation, data were loaded on Cluster 3.0; Log 2 expression ratios were filtered to find genes with a minimum expression ratio of three-fold different from untreated controls. Hence we obtained 1281 differentially expressed genes (651 up-regulated and 630 down-regulated, supplementary information). Hierarchical clustering revealed specific patterns induced by each treatment (Figure 2). In this method of clustering, relationships among objects (genes)are represented by a tree whose branch lengths reflect the degree of similarity between the objects, as assessed by a pairwise similarity function. The computed trees can be used to order genes in the original data table, so that genes or groups of genes with similar expression patterns are adjacent. The ordered table can then be displayed graphically as figure 2, with a representation of the tree to indicate the relationships among genes. Interestingly, most down-regulated genes were co-regulated by either drug, alone or combined. On the contrary, up-regulated genes were somehow specific for each treatment. It was found that 153, 178 and 352 genes were specifically induced 3-fold by hydralazine, valproic acid or the combined treatment respectively. Additional files 1, 2 and 3 list the genes with the highest degree of over-expression that also have a function known. The complete list of genes whose expression changed is provided as supplementary information (additional file 4).

### Effect of cytotoxic drugs in combination with hydralazine and valproic acid in HeLa cells

**Figure 3:**
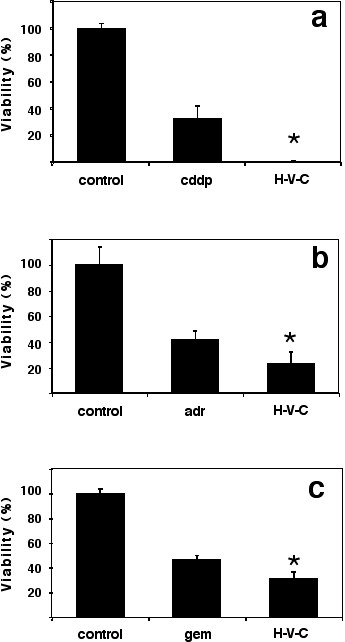
**Effect of cytotoxic drugs in combination with hydralazine and valproic acid in HeLa cells**. HeLa cells were treated for 24 hours with cisplatin, adriamycin or gemcitabine at or close to IC^50 ^plus hydralazine and valproic acid. Afterwards medium was removed and fresh medium containing only hydralazine and valproic acid was added for additional 48 hours after which viability was measured. There was a statistically significant higher cytotoxicity of the chemotherapeutic agent when used in combination with hydralazine plus valproic acid. Viability with cisplatin was 37% and zero after co-treatment with hydralazine-valproic acid (p < 0.002). Corresponding values for adriamycin and gemcitabine alone or with co-treatment were 42% versus 27% (p < 0.020) and 45% versus 37% (p < 0.012) respectively.

HeLa cells were treated for 24 hours with cisplatin, adriamycin or gemcitabine at or close to IC^50 ^plus hydralazine and valproic acid. Afterwards medium was removed and fresh medium containing only hydralazine and valproic acid was added for additional 48 hours after which viability was measured. Figure 3 shows a statistically significant higher cytotoxicity of the chemotherapeutic agent when used in combination with hydralazine plus valproic acid. Cisplatin alone at 12 μM caused a reduction in viability to 37% in HeLa cells which was essentially zero viability when cells were co-treated with hydralazine plus valproic acid (p < 0.002). The increasing toxicity of the combination was also seen with adriamycin and gemcitabine. Viability was 42% and 45% in the chemotherapy drugs alone versus 27% (p < 0.020) and 37% (p < 0.012) respectively, when hydralazine plus valproic acid were added.

### Antitumor evaluation of the combination in vivo

**Figure 4:**
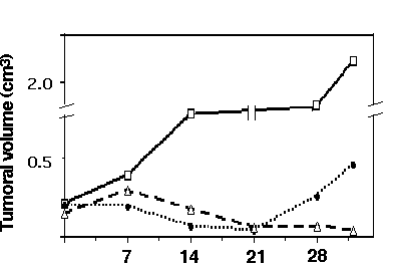
**Antitumor effect of the combination of hydralazine and valproic acid plus adriamycin in a nude mice xenograft model of fibrosarcoma**. Control: solid line with open squares. Tumors in animal treated with adriamycin (dotted line with closed circles) and hydralazine-valproic acid and adriamycin (broken line with open triangles) had their tumors shrinkaged at day 21, however, in animals receiving hydralazine and valproic acid tumors failed to regrowth while in those with only adriamycin tumors increased in size shortly after day 21. Saline, or adriamycin injections were performed at days 7, 14 and 21. In the group with the three-drug combination, hydralazine and valproic acid were administered daily from day 1 (seven days before the first injection of adriamycin (day 7) until sacrifice (day 35).

To investigate whether the hydralazine and valproic acid combination has antitumor effects in vivo, we used a sarcoma xenograft model in nu/nu mice. Animals were treated with adriamycin by the intraperitoneal route with a dose of 2.2 mg/Kg on a weekly basis for 3 weeks, starting when the tumor volume reached at least 1 cm in its larger diameter. Treatment with hydralazine plus valproic acid started the week (day 1) before the first injection of adriamycin (day 7) and continued until animals were sacrificed. Figure 4 shows that in untreated control animals tumor growth increased rapidly whereas in the animals treated with adriamycin with or without hydralazine plus valproic acid, tumors disappeared at day 21. However, there was a rapid regrowth starting at day 21 in the animals treated only with adriamycin, whereas animals treated with hydralazine plus valproic acid remained free of tumor.

## Discussion

Aberrant gene transcription resulting from epigenetic changes, -namely DNA promoter hypermethylation and histone deacetylation- are frequent events in the molecular pathogenesis of malignant transformation [1]. Gene transcription in eukaryotic cells is a highly regulated phenomenon. Although the whole process has several levels of regulation, at the top of these stands the status of chromatin structure which affects the ability of transcriptional regulatory proteins and RNA polymerases to find access to specific promoters and activate gene transcription [19]. DNA hypermethylation and histone deacetylation are critical for determining a closed chromatin structure that may be responsible or related to the aberrant gene transcription in the malignancies, hence its reversal using DNA methylation inhibitors as well as histone deacetylase inhibitors leads to antitumor effects as demonstrated in preclinical [20] and clinical studies [21].

On these basis we wanted to analyze the antineoplastic effect of hydralazine plus valproic acid on a panel of cancer cell lines from diverse origins as epigenetic alterations are common to all tumor types investigated [22]. The results of this study show that valproic acid has a strong inhibitory effect in all cell lines tested and that this effect is statistically significant increased with hydralazine in one of these seven cell lines. However, hydralazine alone at the concentration tested, had no growth inhibitory properties which is not surprising. With exception of 5-aza-2'-deoxycytidine which is a classical cytotoxic agent with demethylating properties [23], the in vivo or in vitro antiproliferative effects of other DNA demethylating agents such as procainamide [24], zebularine [25], tea polyphenol (-)-epigallocatechin-3-gallate [6] and RG108 [26] are low or absent at DNA demethylating concentrations. Contrarily, valproic acid showed a strong inhibitory effect in all cell lines tested as previously seen in a variety of experimental systems [15]. The cytotoxicity of valproic acid can be related to its actions on several targets such as peroxisome proliferator-activated receptor δ (PPAR-δ), deregulation of β-catenin, and the transcriptional induction of the cell cycle inhibitor p21 [16,27-29], however, it seems that its HDAC inhibitory effect is largely responsible for its cytotoxicity [30]. Interestingly, our results show that in the cell line SW480, the combined treatment produced higher cytotoxicity than valproic acid alone as it has been observed in other models combining a DNA demethylating agent and a HDAC inhibitor [31,32]. The growth inhibition of valproic acid has been associated with a G1 or G2/M phase arrest as well as apoptosis in certain cell lines [33,34].

The data on the synergy between a DNA methylation inhibitor and a HDAC inhibitor for gene re-expression [35] led us to explore the effect of hydralazine plus valproic acid on global gene expression. For this analysis we chose the SW480 cancer colon cell line as it was the one that showed higher growth inhibition with the combination of hydralazine and valproic acid (Figure 1). The microarray analysis demonstrated that 1281 (651 up-regulated and 630 down-regulated, 2.33% from 55 000 genes) were differentially expressed at least 3-fold different from an untreated control. Among the up-regulated genes 153 (0.28%) were specifically induced by hydralazine and 178 (0.32%) by valproic acid. However, when cells were exposed 4 days to hydralazine and then 16 hours to valproic acid, the number rose to 352 (0.64%) demonstrating that these epigenomic active drugs have a synergistic effect on gene reactivation. Synergy between a DNA methylation inhibitor 5-aza-2'-deoxycytidine and a deacetylase inhibitor trichostatin A on gene re-expression at a global scale is already known; like to our results it has been shown in another system that the percentage of up-regulated genes with 5-aza-2'-deoxycytidine was 1.9%, and 1.1% with trichostatin A but rose to 10.4% with the combination [36] indicating that the reversal of two epigenetic factors (i.e., DNA demethylation and/or histone hyperacetylation) synergizes gene re-expression [37]. We are currently investigating a number of cancer cell lines to better define the patterns of gene expression induced by these drugs; to determine whether these effects are methylation dependent or independent; and to define whether the synergy for up-regulating genes matches the pattern of growth inhibition observed in the cell lines.

Upon any stimuli, cells must orchestrate a number of changes in the expression of genes as well as other dynamic processes such as protein phosphorylation, protein trafficking, and protein-protein interactions in order to cope with cytotoxic stress [38]. It is therefore vital for cells to have an intact transcription regulation machinery. On this basis we hypothesized that malignant cells exposed to cytotoxic chemotherapeutic agents, regardless of their intrinsic sensitivity to hydralazine, valproic acid or the combination would have a deranged response and perhaps compromised survival if co-treated with these two drugs that affect DNA methylation and histone acetylation. Thus, we chose for this analysis HeLa cells which are not inhibited by hydralazine nor showed increased inhibition to hydralazine and valproic acid in combination (Figure 1). The results showed that indeed, cells exhibited increased cytotoxicity to chemotherapy agents regardless of their mechanism of action since we used cisplatin, a DNA-damaging agent, adriamycin a drug targeting topoisomerase II and the antimetabolite gemcitabine. It is noticeable however, that the cytotoxicity was higher for cisplatin as could be expected because loosening-up the chromatin structure by histone acetylation increases the efficiency of anticancer drugs that target DNA [39]. To further support our hypothesis on the higher antitumor of cytotoxic agents when used concurrently with hydralazine and valproic acid led us to test the combination plus adriamycin in a nude mice model xenografted with HT1080 fibrosarcoma cells, that like HeLa cells had no significantly higher growth inhibition when treated with both hydralazine and valproic acid. The results of higher antineoplastic effect in mice were concordant with the observed in vitro for HeLa cells. Remarkably, while tumors receiving only adriamycin re-grew after the last dose of adriamycin, the animals treated with hydralazine plus valproic acid showed no tumor regrowth. These results suggest that the antitumor efficacy of the combination was higher and therefore no microscopic residual remained or alternatively the treatment disabled the proliferative capacity of cells. This in principle may be particularly relevant for patients who have minimal residual disease after curative attempt by surgery, chemotherapy, and/or radiotherapy.

## Conclusion

Hydralazine and valproic acid two widely used drugs that have DNA methyltransferase and histone deacetylase inhibitory activities respectively have promising antineoplastic effects and the ability to up-regulate a number of genes. In addition these drugs seem to potentiate the effect of chemotherapy.

## Methods

### Cell culture

Cell lines MCF-7 breast adenocarcinoma, HeLa cervical cancer, HT1080 sarcoma, KB laryngeal carcinoma, SW480 colon carcinoma, Hep-2 oral carcinoma and D54 glioma cells were cultured at 37°C in a humidified atmosphere containing 5% CO_2 _in DMEM supplemented with 10% (v/v) fetal calf serum (Life Technologies, Inc.).

### Cytotoxicity assays

Hydralazine and valproic acid alone. Cells were seeded into 96-well microtiter Falcon plates (Becton Dickinson, Franklin Lakes, NJ) at 1.5–2.5 × 10^3 ^cells/well in 0.1 ml of complete medium. The next day, cells were treated for 5 days in complete medium with hydralazine at 10 μM and valproic acid at 1 mM. Medium with drugs was changed every other day. At day 6 cell viability was measured by conventional MTT dye reduction assay. Briefly, 50 μl of 5 mg/ml MTT reagent in PBS were added to each well. Viable cells with active mitochondria reduce the MTT to an insoluble purple formazan precipitate that is solubilized by the subsequent addition of 150 μl of DMSO. The formazan dye was measured spectrophotometrically using an ELISA reader. All assays were performed in triplicate. The cytotoxic effect of each treatment was expressed as a percentage of cell viability relative to untreated control cells (percentage of control) and is defined as [(*A*_570 nm_treated cells)/*A*_570 nm_non-treated cells)] × 100.

Cytotoxicity of chemotherapy plus hydralazine and valproic acid. HeLa cells were seeded into 96-well micro titer Falcon plates (Becton Dickinson, Franklin Lakes, NJ) at 1.5–2.5 × 10^3 ^cells/well in 0.1 ml of complete medium. The next day, cells were treated for 72 hours in complete medium with hydralazine at 10 μM and valproic acid at 1 mM. At day four fresh medium containing hydralazine, valproic acid plus the test drug (cisplatin at 12 μM, gemcitabine at 2 μM and doxorubicin at 30 μM) was added for 24 hours. At day five, cell viability was assayed as above described. The data are the mean values ± SD for *n *= 3. Differences between groups were analyzed using one-way ANOVA test. The critical level of significance was set at *p *<0.05.

### Gene expression

We used the Amersham (Piscataway, NJ) CodeLink system containing 55000 genes probes. This array contains a broad range of genes derived from publicly available, well-annotated mRNA sequences. The CodeLink array is unique in being capable of detecting minimal differences in gene expression, as low as 1.3-fold with 95% confidence, because of the novel three-dimensional aqueous gel matrix, which the empirically tested 30-mer oligonucleotides are deposited on [40].

### Target preparation and microarray hybridization

All reagents were provided in the CodeLink expression assay kit (Amersham, Piscataway, NJ USA), except where noted. SW480 cells were treated with hydralazine at 10 μM for 96 hours; valproic acid for 16 hours; or both: hydralazine for 96 hours then valproic acid was added for additional 16 hours. Total RNA was extracted using trizol reagent. cRNA synthesis was performed as per the manufacturer's instructions using 1 μg of total RNA. First-strand cDNA was generated using Superscript II reverse transcriptase and a T7 primer. Subsequently, second-strand cDNA was produced using *E. coli *DNA polymerase I and RNase H. The resultant double-stranded cDNA was purified on a QIAquick column (Qiagen, Valencia, CA) and cRNA was generated via an in vitro transcription reaction using T7 RNA polymerase and biotin-11-UTP (Perkin-Elmer, Boston, MA). cRNA was purified on a RNeasy column (Qiagen), quantified by UV spectrophotometry, and 10 μg were then fragmented by heating at 94°C for 20 minutes in the presence of magnesium. The fragmented cRNA was hybridized overnight at 37°C in hybridization buffer to a CodeLink Whole Human Genome Bioarray in an Innova 4080 shaking incubator (New Brunswick, Edison, NJ) at 300 rpm.

### Post-hybridization processing and scanning

After hybridization, the arrays were washed in 0.75× TNT buffer at 46°C for 1 hour followed by incubation with Cy5 Streptavidin (Amersham) at room temperature for 30 minutes in the dark. Arrays were then washed in 1× TNT followed by a rinse in 0.05% Tween 20 in water. The slides were then dried by centrifugation and kept in the dark until scanning. Images were captured on ArrayWorx Scanner (Applied).

### Normalization and statistical analysis

Images were analyzed using CodeLink Expression Analysis Software. Expression values were globally normalized to the median expression value of the whole array spots. Samples were normalized against the median of the control sample. To circumvent division by zero, values below background noise were taken as 5. This step also allowed us to manage values with CV below 10% [41]. Finally the normalized expression data were analyzed using Cluster 2.0 [42] and Onto-tools [43].

### Experiments in mice

Three groups of 6 nu/nu 6-week-old female mice (Harlan Teklad) were injected with 6 × 10^6 ^HT1080 cells in the flank. After 4–8 weeks, once the tumors reached a size between 0.5 and 1 cm in diameter, groups were treated as follows: group 1 intraperitoneal injection of 200 μl of normal saline weekly three times; group 2) intraperitoneal injection of adriamycin at 2.2 mg/Kg weekly three times (days 7, 14 and 21) and group 3) intraperitoneal daily injection of both hydralazine at 5 mg/kg, and valproic acid at a total dose of 4 mg starting 7 days before the first application of adriamycin. Daily administration of hydralazine and valproic acid was maintained until sacrifice.

## Competing interests

The author(s) declare that they have no competing interests.

## Authors' contributions

A C-B, C C-L and E R-L performed the in vitro testing; C P-P and C T-B performed the microarray analysis; E P-C, A G-F and B S-P performed the experiments in mice; GC, MC and L T-C contributed in the analysis and discussion of results; A D-G conceived and wrote the manuscript.

## Supplementary Material

Additional File 1 Click here for file

Additional File 2 Click here for file

Additional File 3 Click here for file

Additional File 4 Click here for file
