# Supplementary material for: Antineoplastic effects of the DNA methylation inhibitor hydralazine and the histone deacetylase inhibitor valproic acid in cancer cell lines
Source: Cancer Cell Int. 2006 Jan 31;6:2. doi: 10.1186/1475-2867-6-2 (PMC1408081; doi:10.1186/1475-2867-6-2)
Supplement: Additional File 1 [file 1475-2867-6-2-S1.doc]

## Additional file 1 - Most up-regulated genes with known function induced by hydralazine

| Gene | Unigene ID | Name | Cytoband | Function^a^ |
| --- | --- | --- | --- | --- |
| *CHD6* | Hs.371979 | Chromodomain helicase DNA binding protein 6 | 20q12 | Member of the SNF2/RAD54 helicase family, contains two chromodomains, a helicase domain, and an ATPase domain. |
| *GRIA3* | Hs.377070 | Glutamate receptor, ionotrophic, AMPA 3 | Xq25 | Belongs to a family of AMPA receptors |
| *MGAT5* | Hs.115903 | Mannosyl (alpha-1,6-)-glycoprotein beta-1,6-N-acetyl-glucosaminyltransferase | 2q21 | Involved in the synthesis of protein-bound and lipid-bound oligosaccharides. |
| *NRIP1* | Hs.155017 | Nuclear receptor interacting protein 1 | 21q11.2 | Interacts with the hormone-dependent activation domain AF2 of nuclear receptors. Also known as RIP140, this protein modulates transcriptional activity of the estrogen receptor. |
| *PSCD1* | Hs.191215 | Pleckstrin homology, Sec7 and coiled-coil domains 1(cytohesin 1) | 17q25 | Members of this family appear to mediate the regulation of protein sorting and membrane trafficking. Regulates adhesiveness of integrins at the plasma membrane of lymphocytes. |
| *RGS10* | Hs.501200 | Regulator of G-protein signalling 10 | 10q25 | Regulatory molecule that act as GTPase activating protein (GAPs) for G alpha subunits of heterotrimeric G proteins. |
| *NMNAT1* | Hs.546425 | Nicotinamide nucleotide adenylyltransferase 1 | 1p36 | Involved in hundreds of metabolic redox reactions and are utilized in protein ADP-ribosylation, histone deacetylation, and in some Ca(2+) signaling pathways. |
| *PPP2R5C* | Hs.368264 | Protein phosphatase 2, regulatory subunit B (B56), gamma isoform | 14q32 | Belongs to the phosphatase 2A regulatory subunit B family. Protein phosphatase 2A is one of the four major Ser/Thr phosphatases, and it is implicated in the negative control of cell growth and division. |
| *IL21R* | Hs.210546 | Interleukin 21 receptor | 16p11 | The ligand binding of this receptor leads to the activation of multiple downstream signaling molecules, including JAK1, JAK3, STAT1, and STAT3. |
| *PTPN7* | Hs.402773 | Protein tyrosine phosphatase, non-receptor type 7 | 1q32.1 | PTPs are known to be signaling molecules that regulate a variety of cellular processes including cell growth, differentiation, mitotic cycle, and oncogenic transformation. |
| *ACTR2* | Hs.393201 | ARP2 actin-related protein 2 homolog (yeast) | 2p14 | Major constituent of the ARP2/3 complex. This complex is located at the cell surface and is essential to cell shape and motility through lamellipodial actin assembly and protrusion |
| *ADAM12* | Hs.386283 | A disintegrin and metalloproteinase domain 12 (meltrin alpha) | 10q26.3 | Is a membrane-anchored protein implicated in cell-cell and cell-matrix interactions. |
| *TAS2R16* | Hs.272395 | Taste receptor, type 2, member 16 | 7q31.1 | Member of the G protein-coupled receptor superfamily. |
| *GALNT9* | Hs.301062 | UDP-N-acetyl-alpha-D-galactosamine:polypeptide N-acetylgalactosaminyltransferase 9 | 12q24.33 | Belongs to the GalNAc-Ts family of enzimes, wich initiate mucin-type O-linked glycosylation in the Golgi apparatus by catalyzing the transfer of GalNAc to serine and threonine residues on target proteins. |
| *EPS15* | Hs.83722 | Epidermal growth factor receptor pathway substrate 15 | 1p32 | Involved on the EGFR pathway. The protein is present at clatherin-coated pits and is involved in receptor-mediated endocytosis of EGF. |
| *FBLP-1* | Hs.530101 | Filamin-binding LIM protein-1 | 1p36.13 | This gene product localizes at cell junctions and may link cell adhesion structures to the actin cytoskeleton |
| *FMNL2* | Hs.149566 | Formin-like 2 | 2q23.3 | It has no known function but may have a role in the Wnt signaling pathway. |
| *ADK* | Hs.500118 | Adenosine kinase | 10q22 | Catalyzes the transfer of the gamma-phosphate from ATP to adenosine, thereby serving as a regulator of concentrations of both extracellular adenosine and intracellular adenine nucleotides. |
| *F3* | Hs.62192 | Coagulation factor III (thromboplastin, tissue factor) | 1p22 | Enables cells to initiate the blood coagulation cascades, and it functions as the high-affinity receptor for the coagulation factor VII. |

^a^Function obtained from SOURCE, at http://smd.stanford.edu/cgi-bin/source/sourceSearch
