# Supplementary material for: Antineoplastic effects of the DNA methylation inhibitor hydralazine and the histone deacetylase inhibitor valproic acid in cancer cell lines
Source: Cancer Cell Int. 2006 Jan 31;6:2. doi: 10.1186/1475-2867-6-2 (PMC1408081; doi:10.1186/1475-2867-6-2)
Supplement: Additional File 2 [file 1475-2867-6-2-S2.doc]

**Additional file 2 - Most up-regulated genes with known function induced by valproic acid**

| Gene | Unigene ID | Name | Cytoband | Function^a^ |
| --- | --- | --- | --- | --- |
| CABLES1 | Hs.11108 | Cdk5 and Abl enzyme substrate 1 | 18q11.2 | Cyclin-dependent kinase (CDK)-binding protein that plays a role in proliferation and/or differentiation |
| GRID2 | Hs.480281 | Glutamate receptor, ionotropic, delta 2 | 4q22 | GRID2 belongs to the family of ionotropic glutamate receptors which are the predominant excitatory neurotransmitter receptors in the mammalian brain. Plays a role in neuronal apoptotic death. |
| OR10A2 | Hs.371762 | Olfactory receptor, family 10, subfamily A, member 2 | 11p15.4 | The olfactory receptor proteins are members of a large family of G-protein-coupled receptors (GPCR) arising from single coding-exon genes. |
| OR2A20P | Hs.133517 | Olfactory receptor, family 2, subfamily A, member 20 pseudogene | 7q35 | The olfactory receptor proteins are members of a large family of G-protein-coupled receptors (GPCR) arising from single coding-exon genes. |
| SLC22A16 | Hs.520319 | Solute carrier family 22 (organic cation transporter), member 16 | 6q22.1 | Organic ion transporters, such as SLC22A16, transport various medically and physiologically important compounds, including pharmaceuticals, toxins, hormones, neurotransmitters, and cellular metabolites. These transporters are also referred to as amphiphilic solute facilitators (ASFs |
| PADI3 | Hs.149195 | Peptidyl arginine deiminase, type III | 1p36.13 | Catalyzes posttranslational protein modification by converting arginine to citrulline in the presence of calcium ions. |
| ARHGAP17 | Hs.373793 | Rho GTPase activating protein 17 | 16p12.1 | GTPase-activating proteins stimulate the intrinsic GTP hydrolysis of small G proteins, such as RHOA, RAC1, and CDC42. |
| VAMP4 | Hs.6651 | Vesicle-associated membrane protein 4 | 1q24 | This protein may play a role in trans-Golgi network-to-endosome transport. |
| MEF2A | Hs.268675 | MADS box transcription enhancer factor 2, polypeptide A (myocyte enhancer factor 2A) | 15q26 | The MEF2 genes are members of the MADS gene family (named for the yeast mating type-specific transcription factor MCM1), a family that also includes several homeotic genes and other transcription factors, all of which share a conserved DNA-binding domain |
| PSMB5 | Hs.422990 | Proteasome (prosome, macropain) subunit, beta type, 5 | 14q11.2 | This gene encodes a member of the proteasome B-type family, also known as the T1B family, that is a 20S core beta subunit in the proteasome. |
| PTPRD | Hs.446083 | Protein tyrosine phosphatase, receptor type, D | 9p23 | Member of the protein tyrosine phosphatase (PTP) family. PTPs are known to be signaling molecules that regulate a variety of cellular processes including cell growth, differentiation, mitotic cycle, and oncogenic transformation. |
| PRPH | Hs.37044 | Peripherin | 12q12 | Member of the transmembrane 4 superfamily, also known as the tetraspanin family. Most of these members are cell-surface proteins that are characterized by the presence of four hydrophobic domains. The proteins mediate signal transduction events that play a role in the regulation of cell development, activation, growth and motility. |
| GBA2 | Hs.443134 | Glucosidase, beta (bile acid) 2 | 9p13.3 | This gene encodes a microsomal beta-glucosidase that catalyzes the hydrolysis of bile acid 3-O-glucosides as endogenous compounds. This putative transmembrane protein is thought to play a role in carbohydrate transport and metabolism. |
| SEC31L2 | Hs.18889 | SEC31-like 2 (S. cerevisiae) | 10q24.32 | Unknown function. The protein has moderate similarity to rat VAP1 protein which is an endosomal membrane-associated protein, containing a putative Ca2+/calmodulin-dependent kinase II phosphorylation site. |
| KCNK17 | Hs.162282 | Potassium channel, subfamily K, member 17 | 6p21.1 | Belongs to the superfamily of potassium channel proteins containing two pore-forming P domains. |
| SC65 | Hs.446459 | Synaptonemal complex protein SC65 | 17q21.2 | Initially characterized because it was an autoantigen in cases on interstitial cystitis. |
| ACBD4 | Hs.110298 | Acyl-Coenzyme A binding domain containing 4 | 17q21.31 | The gene product binds to acyl-CoA |
| ARL2L1 | Hs.533086 | ADP-ribosylation factor-like 2-like 1 | 3q11.2 | Participates in protein transport |
| BRE | Hs.258314 | Brain and reproductive organ-expressed (TNFRSF1A modulator) | 2p23.2 | Involved in signal transduction |

^a^Function obtained from SOURCE, at http://smd.stanford.edu/cgi-bin/source/sourceSearch
