# Supplementary material for: Antineoplastic effects of the DNA methylation inhibitor hydralazine and the histone deacetylase inhibitor valproic acid in cancer cell lines
Source: Cancer Cell Int. 2006 Jan 31;6:2. doi: 10.1186/1475-2867-6-2 (PMC1408081; doi:10.1186/1475-2867-6-2)
Supplement: Additional File 3 [file 1475-2867-6-2-S3.doc]

## Additional file 3 - Most up-regulated genes with known function induced by hydralazine and valproic acid

| Symbol | UGCluster | Name | Cytoband | SumFunc |
| --- | --- | --- | --- | --- |
| EMCN | Hs.152913 | Endomucin | 4q24 | Mucin-like sialoglycoproteins interferes with the assembly of focal adhesion complexes and inhibits interaction between cells and the extracellular matrix. |
| EIF2C2 | Hs.449415 | Eukaryotic translation initiation factor 2C, 2 | 8q24 | Belongs to the Argonaute family of proteins which play a role in RNA interference. It may interact with dicer1 and play a role in short-interfering-RNA-mediated gene silencing. |
| HLA-DRA | Hs.520048 | Major histocompatibility complex, class II, DR alpha | 6p21.3 | HLA-DRA is one of the HLA class II alpha chain paralogues. It plays a central role in the immune system by presenting peptides derived from extracellular proteins. |
| MEIS1 | Hs.526754 | Meis1, myeloid ecotropic viral integration site 1 homolog (mouse) | 2p14 | MEIS1 encodes a homeobox protein belonging to the TALE ('three amino acid loop extension') family of homeodomain-containing proteins. |
| HOXA7 | Hs.446318 | Homeo box A7 | 7p15-p14 | Encodes a DNA-binding transcription factor which may regulate gene expression, morphogenesis, and differentiation. |
| SRC | Hs.195659 | V-src sarcoma (Schmidt-Ruppin A-2) viral oncogene homolog (avian) | 20q12-q13 | This gene is highly similar to the v-src gene of Rous sarcoma virus. May play a role in the regulation of embryonic development and cell growth. |
| PADI3 | Hs.149195 | Peptidyl arginine deiminase, type III | 1p36.13 | Catalyzes posttranslational protein modification by converting arginine to citrulline in the presence of calcium ions. |
| COL25A1 | Hs.112925 | Collagen, type XXV, alpha 1 | 4q25 | Carboxy-lyase activity, involved in cell adhesion. |
| TFCP2L3 | Hs.161160 | Transcription factor CP2-like 3 | 8q22.3 | Member of a family of transcription factor genes whose archetype is TFCP2. |
| CD8A | Hs.85258 | CD8 antigen, alpha polypeptide (p32) | 2p12 | Cell surface glycoprotein found on most cytotoxic T lymphocytes that mediates efficient cell-cell interactions within the immune system. |
| DOCK2 | Hs.325528 | Dedicator of cytokinesis 2 | 5q35.1 | Encodes a hematopoietic cell-specific CDM family protein that is indispensable for lymphocyte chemotaxis. |
| SON | Hs.517262 | SON DNA binding protein | 21q22.1 | Binds to a specific DNA sequence upstream of the upstream regulatory sequence of the core promoter and second enhancer of human hepatitis B virus (HBV). The protein shows sequence similarities with other DNA-binding structural proteins such as gallin, oncoproteins of the MYC family, and the oncoprotein |
| ELMO2 | Hs.210469 | Engulfment and cell motility 2 (ced-12 homolog, C. elegans) | 20q13 | Interacts with the dedicator of cyto-kinesis 1 protein. Similarity to a C. elegans protein suggests that this protein may function in phagocytosis of apoptotic cells and in cell migration. |
| PRPH | Hs.37044 | Peripherin | 12q12 | Member of the transmembrane 4 superfamily, also known as the tetraspanin family. Mediate signal transduction events that play a role in the regulation of cell development, activation, growth and motility. |
| PRDM5 | Hs.132593 | PR domain containing 5 | 4q25 | The protein encoded by this gene is a transcription factor of the PR-domain protein family. It contains a PR-domain and multiple zinc finger motifs. Transcription factors of the PR-domain family are known to be involved in cell differentiation and tumorigenesis. |
| SIAT1 | Hs.207459 | ST6 beta-galactosamide alpha-2,6-sialyltranferase 1 | 3q27-q28 | Catalyzes the transfer of sialic acid from CMP-sialic acid to galactose-containing substrates. Is involved in the generation of the cell-surface carbohydrate determinants and differentiation antigens HB-6, CDw75, and CD76. This protein is a member of glycosyltransferase family 29. |
| ADAMTS19 | Hs.23751 | A disintegrin-like and metalloprotease with thrombospondin type 1 motif, 19 | 5q31 | This gene encodes a disintegrin and metalloproteinase with thrombospondin (ADAMTS) motifs-19, which is a member of the ADAMTS protein family. |
| KREMEN1 | Hs.229335 | Kringle containing transmembrane protein 1 | 22q12.1 | This gene encodes a high-affinity dickkopf homolog 1 (DKK1) transmembrane receptor that functionally cooperates with DKK1 to block wingless (WNT)/beta-catenin signaling. The encoded protein is a component of a membrane complex that modulates canonical WNT signaling through lipoprotein receptor-related protein 6 (LRP6). |
| MR1 | Hs.101840 | Major histocompatibility complex, class I-related | 1q25.3 | Belongs to MHC class I protein complex. |

^a^Function obtained from SOURCE, at http://smd.stanford.edu/cgi-bin/source/sourceSearch
